# Differential manifestations of prepubescent, pubescent and postpubescent pediatric patients with systemic lupus erythematosus: A retrospective study of 96 Chinese children and adolescents

**DOI:** 10.1186/1546-0096-10-12

**Published:** 2012-05-02

**Authors:** Li-Lan Chiang, Yu-Tsan Lin, Hung-Yi Chan, Bor-Luen Chiang

**Affiliations:** 1 Department of Pediatrics, National Taiwan University Hospital and National Taiwan University College of Medicine, Taipei, Taiwan; 2 Department of Medical Research, National Taiwan University Hospital, #7 Chung-Shan South Road, Taipei, Taiwan

**Keywords:** Pediatric systemic lupus erythematosus, Age, Pubescent

## Abstract

**Background:**

Children represent 10-20% of all systemic lupus erythematosus (SLE) patients. Their clinical manifestations and outcomes vary with age. We aim to clarify the relationship between pubescent status and the clinical manifestations of pediatric SLE.

**Methods:**

In this study, pediatric SLE patients were divided into three groups, based on age at disease onset (≦8, 8–13 & 13–18 years), defined as prepubescent, pubescent and postpubescent, respectively. Initial clinical manifestations and laboratory characteristics at diagnosis were analyzed.

**Results:**

Ninety-six patients were entered into the study: 8 had disease onset before age 8, while 49 were between 8–13 and 39 of them were 13–18. Female predominance was noted in all three groups (2.5-7.0:1). Postpubescents showed significantly more renal involvement and lymphopenia, along with lower levels of C3 and C4, when compared with prepubescents. They also showed significantly more lymphopenia when compared with pubescents. Pubescents showed significantly more renal involvement, leukopenia and lupus anticoagulant (LAC) positivity, along with lower C3 and C4 levels, when compared with prepubescents. Pubescents also showed significantly higher anti-Sm antibody positivity when compared with postpubescents. Prepubescents showed significantly more splenomegaly and anti-Jo-1 antibody positivity when compared with those of pubescents. The results showed that the disease activity (SLEDAI-2K score) correlated positively with age at disease onset and negatively with disease duration before diagnosis (*p* = 0.011).

**Conclusions:**

Age at disease onset is related to initial manifestations in pediatric SLE patients at our center. Certain parameters such as renal involvement, splenomegaly, low C3 level, low C4 level, lymphopenia, leukopenia, and anti-Sm & anti-Jo-1 antibody were found to be significantly different among the age groups. Renal involvement might be the key symptom that varies with age.

## Background

Systemic lupus erythematosus (SLE) predominantly affects young women who are at a reproductive age. SLE commences in childhood in 10-20% of patients and early-onset SLE before age of five is rare [1-7]. Most childhood studies have largely omitted patients younger than 10 years old [8-12]. Genes, environment, neuroendocrinology, female sex hormone levels and immune dysregulation might contribute to SLE development [13-17].

Clinical manifestations in SLE patients are extremely variable and its course is unpredictable. The clinical course can range from mild to severe and can be potentially life-threatening. SLE that begins at childhood tends to be more severe, with frequent cardiac, pulmonary and renal involvement and is associated with a more aggressive course than those with older age [18]. Common manifestations are nephritis, hematological involvement including anemia, thrombocytopenia, arthritis, central nervous system lupus, and skin involvement. The relative incidence of these manifestations are different in adults and different age groups of children [19]. The correlation between gender, ethnicity, pubescent status, or age at disease onset and the manifestations and outcomes in children with SLE is well delineated [18-20].

The purpose of this study is to investigate the correlations between pubescent status, age at disease onset, and the manifestations and outcomes of pediatric SLE. We compared the initial clinical manifestations and laboratory findings of pediatric SLE patients in prepubescent, pubescent and postpubescent groups to determine whether or not pubescent status correlates with the above variables and outcomes.

## Methods

### Study design and patient selection

Patients from the Department of Pediatrics, National Taiwan University Hospital, who met the criteria for SLE, from 1975–2010, were reviewed. SLE criteria were utilized in accordance with the American College of Rheumatology (ACR) 1997 revisions [21,22].

Patients were divided into three groups, based on age at disease onset. Group A included patients ≦8 years old (prepubescent), while group B patients were 8–13 (pubescent) and group C patients were 13–18 (postpubescent) [23,24]. Initial clinical manifestations and laboratory findings were recorded during diagnosis.

### Data collection

Age, sex, disease duration before diagnosis, initial clinical presentation, laboratory findings, family (1^st^ & 2^nd^ degree relatives) autoimmune history and Systemic Lupus Erythematosus Disease Activity Index 2000 (SLEDAI-2K) scores upon diagnosis were all noted [25]. The definition of disease onset is the onset of disease symptoms related to SLE criteria. The disease duration before diagnosis is the period from when symptoms first began to the time the child met ACR criteria for SLE.

Mucocutaneous system involvement included malar rash, discoid rash, oral ulcer, photosensitivity and alopecia. Musculoskeletal system involvement included myalgia that resolved with steroid therapy, autoimmune myositis proven by muscle biopsy, and arthritis. Arthritis was defined as the involvement of two or more joints, characterized by tenderness and swelling. Reticuloendothelial system involvement included splenomegaly, lymphadenopathy, hepatomegaly and autoimmune hepatitis, proven by liver biopsy. Gastrointestinal system involvement entailed severe abdominal pain that resolved with steroid therapy. Renal involvement included the presence of proteinuria (>0.5 g/day or 3-plus in urine examination or cellular casts). The renal biopsy was classified by World Health Organization (WHO) criteria (six categories) [26]. Central nervous system involvement included seizure, psychosis and vasculitis, identified by imaging. Serositis included pleural effusion, pericardial effusion, ascites, and peritonitis.

Laboratory thresholds for SLE included leukopenia (<4000/mm^3^), lymphopenia (<1500/mm^3^) and thrombocytopenia (<100,000/mm^3^). Complement fractions, C3 and C4, were considered abnormal if they were below two standard deviations. Antinuclear antibody (ANA) titer and anti-double stranded DNA (anti-dsDNA) antibody level were considered positive when the former was >1:160 and the latter was >75 IU/ml, as determined by enzyme immunoassay (EIA). Anti-cardiolipin antibodies (aCLs) and anti-extractable nuclear antigens (anti-ENAs) were assayed by enzyme-linked immunosorbent assay (ELISA). Lupus anti-coagulant (LAC) was detected through activated partial thromboplastin time, the kaolin clotting time, and the dilute Russell’s viper venom time. We also used Systemic Lupus Erythematosus Disease Activity Index 2000 (SLEDAI-2K) scores as a measure of disease activity at diagnosis.

### Outcomes

Mortality was defined as death. All survivors were followed-up from disease onset until May 2010. Morbidity as renal disease requiring dialysis and renal transplant were also recorded.

### Statistical analysis

Descriptive statistics were used for initial clinical manifestations and laboratory data. We used Pearson’s Chi-squared test to compare categorical variables between the three groups and Mann–Whitney U-testing or Kruskal Wallis test for continuous variables. Spearman’s correlation coefficients were used to calculate the correlation between disease duration before diagnosis and disease activity at diagnosis. Kaplan-Meier survival analysis was used for outcome evaluation. Differences were considered significant for *p*-values < 0.05.

## Results

### Patients

Initial clinical manifestations and laboratory findings are summarized in Table 1. Out of 96 total patients, 74 were female (77.1%). Prepubescents (group A) included 8 patients, while there were 49 pubescents (group B) and 39 postpubescents (group C). The female-to-male (F:M) ratio was 7:1 in group A, 2.5:1 in group B and 4.6:1 in group C. There was no significant difference in female prevalence among the three groups.

**Table 1:** Initial clinical manifestations and laboratory characteristics of pediatric SLE patients of different pubescent age groups

Characteristics	Group A(n = 8)	Group B (n = 49)	Group C (n = 39)	Significance
Mean onset age: years (range)	6.8 (4.7-7.9)	11(8.8-12.9)	14.6(13–17.9)	
Gender (F:M)	7:1	2.5:1	4.6:1	ns
Positive family history (%)	1(12.5)	7 (14.3)	7 (18)	ns
Mean disease duration before diagnosis: months (range)	21.3 (0.8-90)	9.2 (0.1-96)	4.4 (0.3-24)	ns
Organ involvement (%)				
Mucocutaneous	6 (75)	35 (71.4)	29 (74.4)	ns
Malar rash	5 (62.5)	26 (53.1)	19 (48.7)	ns
Discoid rash	1 (12.5)	3 (6.1)	2 (5.1)	ns
Oral ulcer	4 (50)	19 (38.8)	12 (30.8)	ns
Photosensitivity	2 (25)	6 (12.2)	3 (7.7)	ns
Alopecia	0 (0)	7 (14.3)	7 (18)	ns
Musculoskeletal	5 (62.5)	26 (53.1)	21 (53.9)	ns
Myalgia	1 (12.5)	3 (6.1)	4 (10.3)	ns
Arthritis	4 (50)	24 (49)	19 (48.7)	ns
Reticuloendothelial system	3 (37.5)	6 (12.2)	6 (15.4)	ns
Lymphadenopathy	2 (25)	4 (8.2)	3 (7.7)	ns
Splenomegaly	1 (12.5)	0 (0)	2 (5.1)	ns
Hepatic	1 (12.5)	2 (4.1)	1 (2.6)	ns
Gastrointestinal	1 (12.5)	2 (4.1)	3 (7.7)	ns
Renal	1 (12.5)	24 (49)	23 (59)	*p* = 0.01
WHO class I	0/0 (0)	0/12 (0)	0/8 (0)	ns
WHO class II	0/0 (0)	1/12 (8.3)	2/8 (25)	ns
WHO class III	0/0 (0)	4/12 (33.3)	0/8 (0)	ns
WHO class IV	0/0 (0)	9/12 (75)	5/8 (62.5)	ns
WHO class V	0/0 (0)	4/12 (33.3)	1/8 (12.5)	ns
WHO class VI	0/0 (0)	0/12 (0)	1/8 (12.5)	ns
Central nervous system	0 (0)	3 (6.1)	1 (2.6)	ns
Serositis	0 (0)	5 (10.2)	9 (23.1)	ns
Pleural effusion	0 (0)	4 (8,2)	6 (15.4)	ns
Pericardial effusion	0 (0)	2 (4.1)	5 (12.8)	ns
Ascites/Peritonitis	0 (0)	2 (4.1)	4 (10.3)	ns
Hematologic	6 (75)	38 (77.6)	34 (87.2)	ns
Anemia	6 (75)	34 (69.4)	30 (76.9)	ns
Leukopenia	0 (0)	17 (34.7)	13 (33.3)	ns
Lymphopenia	1 (12.5)	18 (36.7)	25 (64.1)	*p* = 0.005
Thrombocytopenia	1 (12.5)	9 (18.4)	12 (30.8)	ns
Laboratory (%)				
Low C3	3 (37.5)	41 (83.7)	34 (87.2)	*p* = 0.004
Low C4	3 (37.5)	42 (85.7)	34 (87.2)	*p* = 0.03
Autoantibodies positive :number (%)				
ANA	8 (100)	46 (93.9)	36 (92.3)	ns
Anti-dsDNA antibody	5 (62.5)	44 (89.8)	30 (76.9)	ns
aCL	0/2 (0)	4/12 (33.3)	3/18 (16.7)	ns
LAC	0/2 (0)	1/13 (7.7)	1/18 (5.6)	ns
Anti-ENA				
Anti-RNP	3/6 (50)	10/30 (33.3)	4/29 (13.8)	ns
Anti-Jo-1	1/6 (16.7)	0/30 (0)	0/29 (0)	*p* = 0.012
Anti-Scl-70	0/6 (0)	3/30 (10)	1/29 (3.4)	ns
Anti-Ro	4/6 (66.6)	10/30 (33.3)	11/29 (37.9)	ns
Anti-La	2/6 (33.3)	4/30 (13.3)	3/29(10.3)	ns
Anti-Sm	1/6 (16.7)	10/30 (33.3)	0/29 (0)	*p* = 0.009

All patients were ethnic Chinese. A family history of autoimmune disease was noted in 12.5% of prepubescents, 14.3% of pubescents and 18% of postpubescents. Mean age at disease onset was 6.8 years (range: 4.7-7.9) in group A, 11 (8.8 -12.9) in group B and 14.6 (13–17.9) in group C.

**Figure 1:**
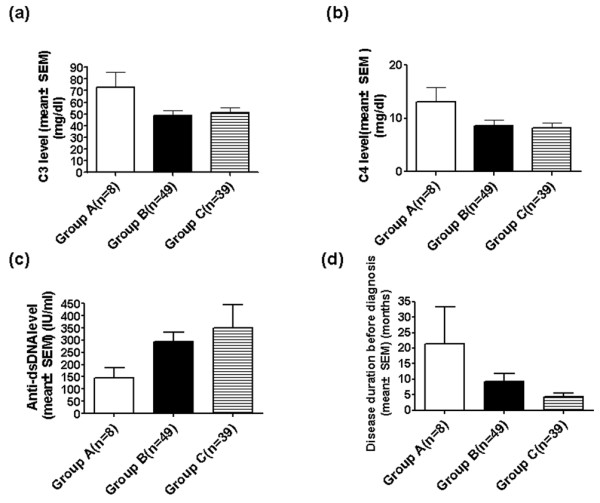
**Levels of C3, C4 & anti-dsDNA antibody, and disease duration before diagnosis, for pediatric SLE patients.** Levels of C3, C4 & anti-dsDNA antibody, and disease duration before diagnosis, for pediatric SLE patients at prepubescent, pubescent and postpubescent ages. **(A)** Mean C3 level was 72 mg/dl in group A, 48 mg/dl in group B and 50 mg/dl in group C. **(B)** Mean C4 level was 13 mg/dl in group A, 8.6 mg/dl in group B and 8.2 mg/dl in group C. **(C)** Mean anti-dsDNA antibody level was 145 IU/ml in group A, 295 IU/ml in group B and 349 IU/ml in group C. **(D)** Mean disease duration before diagnosis was 21.3 months in group A, 9.2 months in group B, and 4.4 months in group C.

Disease duration before diagnosis was defined as the time interval between disease onset and diagnosis. Mean values were 21.3 months (range: 0.8-90) for group A, 9.2 months (0.1-96) for group B and 4.4 months (0.3-24) for group C. In prepubescents, mean disease duration before diagnosis was 2.3-4.8 times longer than values seen for groups B and C (Figure 1).

### Initial clinical manifestations

There was no significant difference in mucocutaneous, musculoskeletal, reticuloendothelial (lymphadenopathy, hepatosplenomegaly and autoimmune hepatitis), gastrointestinal, and central nervous system involvement among the three groups. More than half of the patients had mucocutaneous, musculoskeletal and hematological involvement in each group (Table 1). Gastrointestinal and central nervous system involvement were not common in each group (Table 1).

Renal involvement was present at diagnosis in 23 group C patients (59%) and its frequency significantly increased with age (12.5% in group A, 49% in group B) (*p* = 0.009, A vs. B; *p* = 0.002, A vs. C) (Table 2). Twenty patients underwent renal biopsy; class IV lupus nephritis was the most common in groups B (75%) and C (62.5%). Six patients (30%) had mixed lupus nephritis. One group C patient presented with mixed Class II and V lupus nephritis. Three patients had mixed Class III and IV and two had mixed Class IV and V lupus nephritis in group B. We used the same criteria for deciding whether to perform a renal biopsy: 1) When patients met protein excretion greater than 500 mg/day; or 2) Had an active urinary sediment with hematuria (five or more red blood cells per high power field, most of which are dysmorphic) and often pyuria and cellular casts. Due to the conservative attitude and culture of Chinese patients, some patients rejected the renal biopsy and sometimes the patient’s condition itself did not allow renal biopsy.

**Table 2:** Differential manifestations in prepubescent, pubescent and postpubescent ages for pediatric systemic lupus erythematosus

					*p*-value	
Mean onset age	Group A (n = 8)	Group B(n = 49)	Group C(n = 39)	A vs. B	B vs. C	A vs. C
Renal	1 (12.5)	24 (49)	23 (59)	0.009	ns	0.002
Lymphopenia	1 (12.5)	18 (36.7)	25 (64.1)	ns	0.011	0.007
Low C3	3 (37.5)	41 (83.7)	34 (87.2)	0.004	ns	0.002
Low C4	3 (37.5)	42 (85.7)	34 (87.2)	0.018	ns	0.015
Anti-Sm	1/6 (16.7)	10/30 (33.3)	0/29 (0)	ns	0.001	ns
Anti-Jo-1	1/6 (16.7)	0/30 (0)	0/29 (0)	0.039	ns	ns
Leukopenia	0 (0)	17 (34.7)	13 (33.3)	0.047	ns	ns
Splenomegaly	1 (12.5)	0 (0)	2 (5.1)	0.013	ns	ns
LAC	0/2 (0)	1/13 (7.7)	1/18 (5.6)	0.044	ns	ns

Neurological involvements were noted in three group B patients; manifestations included seizure and psychosis. One patient in group C had a seizure. Although neurological involvement seemed more frequent in group B, the difference among all three groups did not reach statistical significance. Splenomegaly was significantly more common in group A than B (*p* = 0.013) (Table 2).

Manifestations that became more frequent with age included renal involvement, serositis, alopecia, and hematological involvement. Differences in renal involvement among the three groups were also significant (*p* = 0.01) (Table 1).

**Figure 2:**
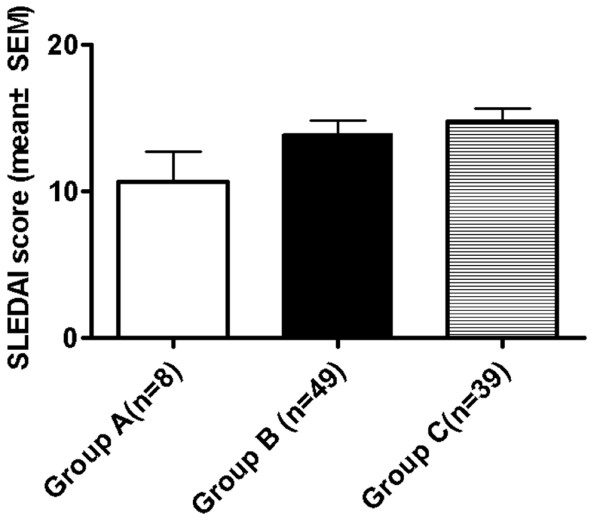
**Mean SLEDAI score at diagnosis for pediatric SLE patients.** Mean SLEDAI score at diagnosis for pediatric SLE patients at prepubescent, pubescent and postpubescent ages. Mean score was 10.6 in group A, 13.8 in group B and 14.7 in group C.

Cumulative disease activity at diagnosis was measured using the SLEDAI score. The mean score was 10.6 (range: 2–20) in group A, 13.8 (4–30) in group B and 14.7 (4–28) in group C. Mean SLEDAI score increased with age, though the differences were not significant (Figure 2).

### Laboratory data

Lymphopenia was present at diagnosis in 25 group C patients (64.1%); its frequency increased with age (12.5% in group A, 36.7% in group B). Group C had a significantly higher prevalence of lymphopenia than the other two groups. (*p* = 0.011 for B vs. C, *p* = 0.007 for A vs. C) (Table 2). Low C3 levels were noted at diagnosis in 34 group C patients (87.2%) and their frequency also increased with age (37.5% in group A, 83.7% in group B). Groups B and C showed significantly higher prevalence of low C3 levels when compared to group A (*p* = 0.004 for A vs. B, *p* = 0.002 for A vs. C). C4 levels showed a similar prevalence (Table 2); groups B and C also showed significantly higher prevalence of low C4 levels when compared with group A (*p* = 0.018 for A vs. B, *p* = 0.015 for A vs. C). Group B anti-Sm antibody positivity (33%) was significantly more prevalent when compared with group C (0%) (*p* = 0.001) while anti-Jo-1 antibody positivity was significantly more prevalent in group A (16.7%) when compared with group B (0%) (*p* = 0.039) (Table 2). Leukopenia and LAC positivity were significantly more prevalent in group B than group A (*p* = 0.047 &*p* = 0.044, respectively). Group A had a significantly higher prevalence of splenomegaly when compared to group B (*p* = 0.013) (Table 2). No statistical differences were found in ANA titer, anti-dsDNA antibody level and aCL positivity. Low levels of C3, C4 and high level of anti-dsDNA antibodies correlated with higher disease activity. Compared with the mean level of C3, C4 and anti-dsDNA antibody of each group, we also found that the disease activity increased with age (Figure 1 A-C). This result was compatible with the increasing mean SLEDAI score of each group (Figure 2).

### Outcomes

**Figure 3:**
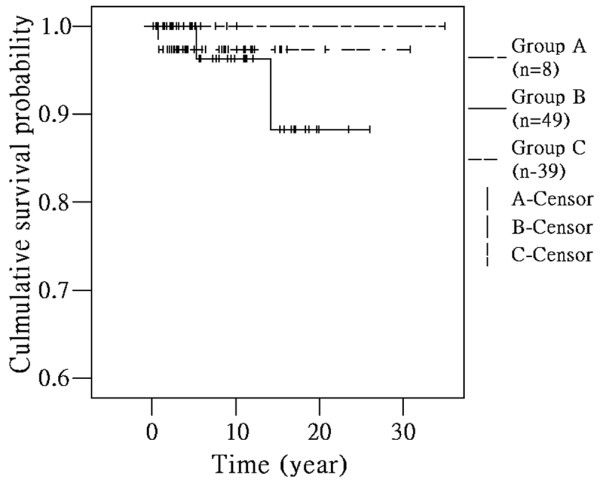
**Kaplan-Meier analysis for survival rates of pediatric SLE patients.** Kaplan-Meier analysis for survival rates of pediatric SLE patients at prepubescent, pubescent and postpubescent ages. Survival rate was 100% in group A, 88.3% in group B and 97.4% in group C.

All surviving patients were followed-up from disease onset until May 2010. Mortality was defined as death. Mean follow-up was 9.7 years (range: 2.4-35.0) in group A, 8.3 (0.1-26.0) in group B and 7.5 (0.5-30.8) in group C. The survival rate was 100% in group A, 88.3% in group B and 97.4% in group C (Figure 3). Two patients died in group B; causes of death were combined renal failure, respiratory failure and pneumonia in one patient while the other had a severe respiratory syncytial virus infection. One patient died in group C due to severe serositis and renal failure. Seven patients were lost to follow-up over one year in group C and five patients in group B. With regards to morbidity, eight (16.3%) patients required dialysis in group B and four (10.2%) in group C. Three of the eights patients requiring dialysis received renal transplant in group B. There was no significant differences noted among the three groups in these mortality and morbidity findings.

## Discussion

Early-onset SLE before the age of five is rare [3]; similarly this early onset occurred in only one of our patients (4.7 years old) while eight (8.3%) had onset before the age of eight. In childhood, girls are affected 4.5 times more often than boys, although the overall ratio varies with age at onset [27-31]. A previous study showed the female predominance is not striking before puberty [20]. The significant role played by sex hormone in the female preponderance was more often noted in postpubescents than prepubescents [18]. The same study also suggested that the greater prevalence of family autoimmune history seen with patients under the age of two years stems from genetic predisposition [18]. However, in the present study, the female-to-male (F:M) ratio was 7:1 in prepubescents, 2.5:1 in pubescents and 4.6:1 in postpubescents. Prepubescents were 1.5-2.8 times more likely to be female than postpubescent and pubescents. The highest frequency of positive family history was seen in the postpubescent group (18%), while in prepubescents and pubescents were 12.5% and 14.3%, respectively. These differences may be affected by many factors, including study method, ethnic diversity, pubescent status and different age cutoff values of the patients compared with the previous studies [18-20].

Age at onset for pediatric SLE patients is related to the expression of the disease in terms of initial clinical manifestations, serological findings, and disease activity [32-35]. We found no significant difference in the incidence of mucocutaneous, musculoskeletal, reticuloendothelial (lymph nodes, hepatosplenomegaly and autoimmune hepatitis) and central nervous system involvement. In contrast, renal involvement was more prevalent in pubescents and postpubescents than prepubescents; indeed, this may be the key symptom that varies with age. The incidence of lymphopenia also increased with age, while the incidence of low C3 and C4 levels increased generally. Marked lymphopenia is significantly related to complement decrease [36]. Hypocomplementemia and positivity for anti-dsDNA antibody, besides being helpful as diagnostic aides, associated more with presence of renal involvement, though the correlation was not statistically significant [19]. In our study, hypocomplementemia significantly correlated with the renal involvement (*p* = 0.017 C3/0.031 C3 or C4). We found that the trend of incidence of renal involvement, lymphopenia, low C3 and C4 level were similar. However, the real mechanisms remain unclear.

It has been shown that changes in the levels of sex hormones led to a number of changes in both innate and adaptive immune cells, affecting antigen presentation, numbers of T and B cells, and antibody secretion [37]. In our study, anti-Sm antibody positivity was significantly greater in pubescents than that of postpubescents. Anti-Jo-1 antibody positivity was significantly greater in prepubescents compared to that of pubescents. LAC positivity was significantly more prevalent in pubescents than prepubescents. According to our results, we suggest that pubescent status may affect different prevalence of autoantibody in three groups. However, more studies are needed to clarify the role of sex hormones in the generation of these autoantibodies.

The correlation of pubescent status and age at disease onset on the disease activity and the outcome in children with SLE is controversial [38]. Early onset disease has been suggested to have higher disease activity and mortality [18-20]. Our results demonstrated that mean SLEDAI score at onset of SLE was higher with increased age of onset. The mean SLEDAI score was compatible with mean C3, C4 levels and anti-dsDNA antibody levels of each group (Figures 2,1 A-C). Mean disease duration before diagnosis was shorter with decreased age of onset; the higher activity of the disease, the shorter the duration before diagnosis (Figures 2,1 D). Disease activity positively correlates with age at disease onset and negatively correlates with disease duration before diagnosis (*p* = 0.011) (data not shown). The survival rate was 100% in prepubescents, 88.3% in pubescents and 97.4% in postpubescents (Figure 3). These effects might be due to the greater prevalence of some major organs involvement such as renal, hematological involvement, and serositis, along with higher activity causing the survival rate to decrease with pubescents and postpubescents.

Renal failure, respiratory failure, infection and serositis contributed to the major causes of death in our patients. Two-third of patients that expired had received alternative treatment such as herbal medications and qigong and had stopped taking their original medications for six months. The outcome of SLE is dependent on early diagnosis and treatment, and, most importantly, patients’ willingness to accept the diagnosis and the need to adhere to advice about therapy [39]. Thus, poor adherence may contribute to the poor outcome in our patients. Sometimes ethnicity is a challenge in the treatment of our patients, because patients of Asian origin are generally quite concerned about the side effects of medicines used for the treatment of SLE. They also felt that medicines in general were harmful and overused, believing that traditional or other non-medical treatments could be quite effective. Communication and education are also important issues that may affect the outcome in our patients.

Several studies have reported that children and adolescents often have an aggressive and severe clinical course and more frequent renal involvement as compared to adults. In our study, we made it clear that the prevalence of renal involvement were significantly different in subgroups of children. The pubescents and postpubescents showed significantly higher prevalence of renal involvement along with higher disease activity than prepubescents. Eight (16.3%) patients required dialysis in pubescents and four (10.2%) in postpubescents. The percentage of renal dialysis is high in pubescents and postpubescents and another study showed about 6.5% of the patients over age 5 requiring renal dialysis [19].

With regards to the limitations of our study, the first was that there was missing data in our retrospective study. Patients who almost met but failed to meet the criteria of SLE had to be excluded from our study. As a result, this had an impact on the number of patients entered into our study and the results. In addition, there were only eight patients in the prepubescent group; more patients might be needed to make statistical analysis more reliable. Another limitation was that we did not have access to detailed information about the SLE treatment of patients which has certainly changed with time. Treatment changes may have affected the mortality rate. Further, the sex hormone profile and the age of menarche were not recorded, so we could not fully clarify the role of sex hormone. The Tanner stage of each patient was also not recorded to evaluate the physical status of puberty. All patients were from an ethnic Chinese population and implications for other ethnic groups is not clear. As the definition of disease onset was from when symptoms related to SLE criteria first began so, this introduces some bias. This self-reporting bias of the timing of disease onset existed and it also likely affected the disease duration before diagnosis.

## Conclusions

In our population of SLE children and adolescents in Taiwan, age at disease onset is related to initial manifestations in pediatric SLE patients. Certain parameters such as renal involvement, splenomegaly, low C3 level, low C4 level, lymphopenia, leukopenia, and anti-Sm & anti-Jo-1 antibody were found to be significantly different among the age groups, while SLEDAI score at onset of SLE was higher with increased age of onset. Renal involvement occurred in approximately 50% of our patients over the age of eight and 10-16% of them needed dialysis in their life. Renal involvement is critical and needs close monitoring. As seen in other populations, adherence to medications played a critical role in the survival rate of our SLE patients.

## Abbreviations

SLE: Systemic lupus erythematosus; LAC: Lupus anticoagulant; SLEDAI-2K: Systemic Lupus Erythematosus Disease Activity Index 2000; WHO: World Health Organization; ANA: Antinuclear antibody; anti-dsDNA: Anti-double stranded DNA; EIA: Enzyme immunoassay; aCLs: Anti-cardiolipin antibodies; anti-ENAs: Anti-extractable nuclear antigens; ELISA: Enzyme-linked immunosorbent assay.

## Competing interests

The authors declare that they have no competing interests.

## Authors’ contributions

Li-Lan Chiang participated in the design of the study and wrote the manuscript.

Yu-Tsan Lin performed the statistical analysis.

Hung-Yi Chan helped to draft the manuscript.

Bor-Luen Chiang participated in the design of the study and helped to draft the manuscript. All authors read and approved the final manuscript.

## Authors’ information

Li-Lan Chiang, M.D

Department of Pediatrics, National Taiwan University Hospital

Yu-Tsan Lin, M.D., Ph.D.

Department of Pediatrics, National Taiwan University Hospital

Hung-Yi Chan, M.D

Department of Pediatrics, National Taiwan University Hospital

Bor-Luen Chiang, M.D., Ph.D.

Department of Pediatrics, National Taiwan University Hospital
